# Novel vehicle detection system based on stacked DoG kernel and AdaBoost

**DOI:** 10.1371/journal.pone.0193733

**Published:** 2018-03-07

**Authors:** Hyun Ho Kang, Seo Won Lee, Sung Hyun You, Choon Ki Ahn

**Affiliations:** 1 School of Electrical Engineering, Korea University, Seoul, Korea; 2 Department of Automotive Convergence, Korea University, Seoul, Korea; Chongqing University, CHINA

## Abstract

This paper proposes a novel vehicle detection system that can overcome some limitations of typical vehicle detection systems using AdaBoost-based methods. The performance of the AdaBoost-based vehicle detection system is dependent on its training data. Thus, its performance decreases when the shape of a target differs from its training data, or the pattern of a preceding vehicle is not visible in the image due to the light conditions. A stacked Difference of Gaussian (DoG)–based feature extraction algorithm is proposed to address this issue by recognizing common characteristics, such as the shadow and rear wheels beneath vehicles—of vehicles under various conditions. The common characteristics of vehicles are extracted by applying the stacked DoG shaped kernel obtained from the 3D plot of an image through a convolution method and investigating only certain regions that have a similar patterns. A new vehicle detection system is constructed by combining the novel stacked DoG feature extraction algorithm with the AdaBoost method. Experiments are provided to demonstrate the effectiveness of the proposed vehicle detection system under different conditions.

## Introduction

Rear-end collisions represent a substantial proportion of all car accidents [1]. Driver assistance systems (DAS) such as forward collision warnings (FCW) and autonomous emergency braking (AEB) using vehicle detection [2], dynamics control and velocity prediction methods [3–7] take active safety systems to prevent car accidents before they happen. The vehicle detection algorithm is an important aspect of all such systems because it is the underlying technology on which they rely [8–9]. Many research studies have used or combined various sensors such as lidar, radar, and vision sensors to increase the precision with which preceding vehicles can be detected [10]. However, the vision sensor both costs less than a system using lidar and radar, and has the advantage that detected objects can be identified with specificity (e.g., as a pedestrian, vehicle, or obstacle). In this paper, we focus on an algorithm that shows highly accurate performance using low-cost vision sensors that can also improve the performance of fusion systems that include lidar and radar.

Detection methods that use optical sensors are divided into appearance- and classifier-based approaches. Appearance-based techniques use specific characteristics of a vehicle's image for detection, such as its symmetry and edges. Most vehicles' rear view mirrors are symmetrical over the vertical center line; thus, it is possible to recognize the locations of vehicles in an image by detecting regions with high horizontal symmetry [11–12].

In addition, most vehicles have horizontal and vertical edges that may be useful for detecting the vehicle's location in an image [13–17]. Classifier-based methods are also used, since appearance-based approaches are easily influenced by the outlying edges of vehicles. Classifiers learn the characteristics of vehicles’ appearances from training images. Common classification schemes for vehicle detection include neural networks [18], the support vector machine (SVM) [19–22] and AdaBoost [23–24]; the AdaBoost approach is the most common of these schemes. A cascade of strong classifiers that consists of several weak classifiers is composed in the training process by automatically classifying positive and negative samples from the detection results and reusing such samples in the AdaBoost training system [25–26]. These algorithms improve system adaptability and the accuracy with which it deals with novel vehicle types and unfamiliar environments. However, there are several drawbacks when we apply the AdaBoost method to a vehicle detection system. First, a preceding vehicle cannot be recognized if the shape of vehicle deviates from the boundary in the training data. Second, when the patterns of vehicles are not visible in images due to the lighting conditions of road, vehicles cannot be detected by the AdaBoost algorithm. In addition, the precision drops sharply when preceding vehicles are distant from the optical sensor because shapes and patterns of vehicle are not visible in such situations. Thus, developing a novel vehicle detection algorithm that can overcome the weaknesses of vehicle detection systems using the AdaBoost method is desirable.

In this paper, a stacked Difference of Gaussian (DoG)–based feature extraction algorithm with AdaBoost is proposed. The proposed algorithm is for vehicles that exist in the ego lines. The algorithm recognizes the shadow and rear wheels beneath the preceding vehicle by applying the stacked DoG kernel with a convolution method and investigating the greatest value of filtering response, which all vehicles have no matter the type or the road environment. This method does not depend on appearance, but rather on the common features all vehicles share. Thus, the proposed algorithm can still detect vehicles with shapes that are distinct from the training data. In addition, even if the patterns outlining vehicles are not visible in the frames due to the road conditions, the correct location of preceding vehicles can still be recognized. This proposed stacked DoG-based feature extraction algorithm is combined with the original cascade strong AdaBoost classifier to create a new system for improved vehicle detection. The performance of new vehicle detection system is demonstrated through comparisons of precision under some harsh environments (e.g., distinct shape of vehicles, vehicles loading cargos, vehicles with no visible patter, or vehicles in tunnels). Through the experimental results, it can be demonstrated that the problems of vehicle detection systems can be overcome better than by using AdaBoost-based algorithm.

This paper is organized as follows: Section II provides the new vehicle detection system, Section III provides the proposed feature extraction algorithm, Section IV includes experiments with various road conditions, and conclusions are stated in Section V.

## Proposed vehicle detection system

**Fig 1:**
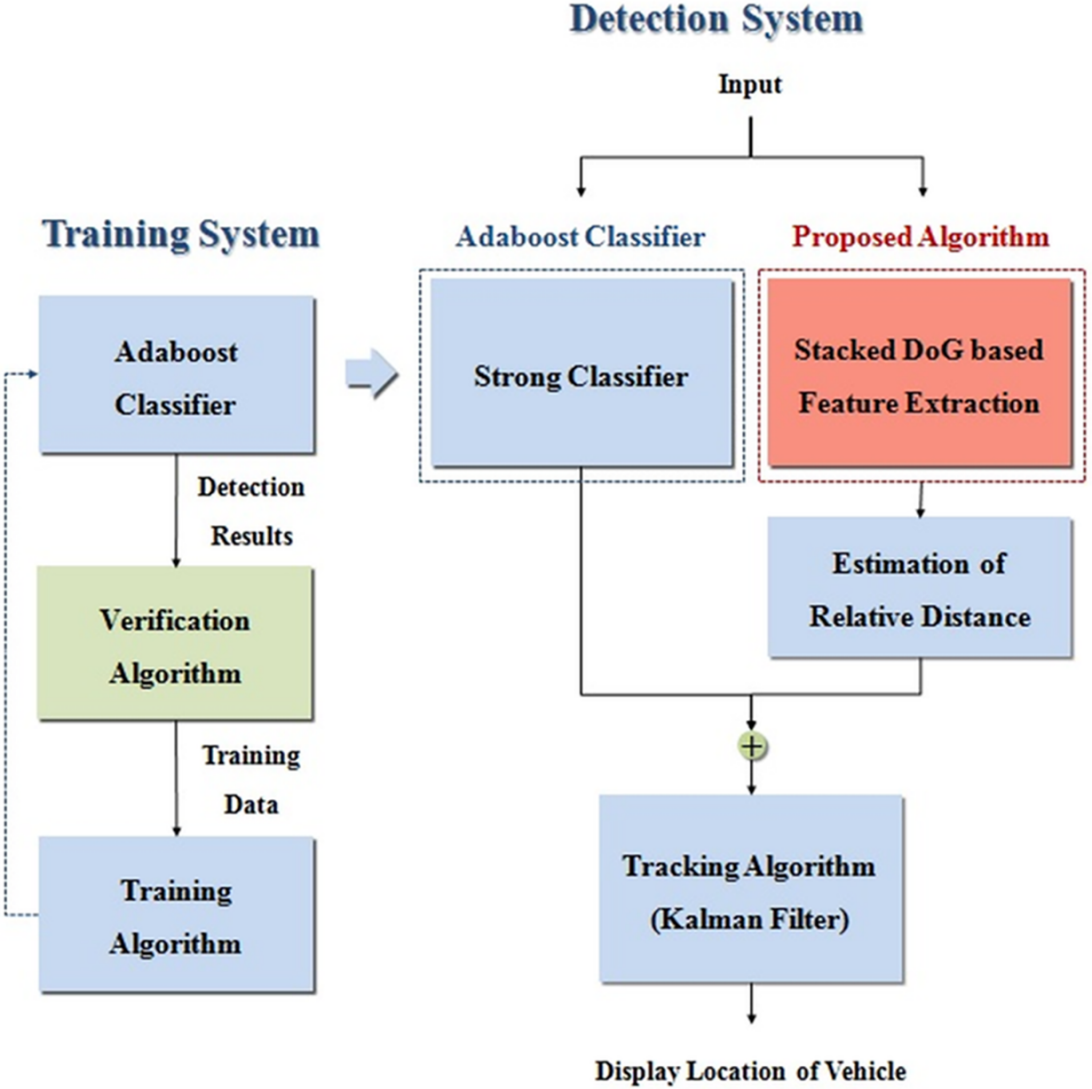
Structure of the proposed vehicle detection system.

As shown in Fig 1, all new vehicle detection systems are composed of training and detection systems. The output of the training system is an AdaBoost strong classifier through a learning algorithm. Sample data is automatically identified by a verification algorithm in the training system as either positive or negative [26]. A detailed structure of the training system is given in Section 2.1. In the detection system, frames are input parallel to the AdaBoost classifier and the proposed stacked DoG-based feature extraction algorithm. The detection results from these two methods are combined and continuously displayed in the image through the tracking algorithm. Section 2.2 provides the whole structure of detection system.

### Training system

The training system utilizes the adaptive boosting method. This AdaBoost training algorithm learns the characteristics of vehicles’ appearances from the sample data, and training is based on a supervised learning approach that utilizes a large set of labeled positive and negative samples [24]. In typical training systems, training samples are identified by human operators as either positive or negative; however, such actions can be performed autonomously by a verification algorithm [25] composed of two steps. Each step presupposes that vehicles have a complex pattern and symmetrical appearance in image frames. Thus, if a sample has a simple pattern, it cannot be identified as a vehicle in the first step. The measure of the degree of symmetry can be detected in the second step, but if an image sample does not appear to be left–right symmetric, it cannot be identified as a vehicle. The measures of the degree of complexity and symmetry in an image are defined as equations [25]. Thus, approximately 5,000 positive samples and 10,000 negative samples can be obtained using this verification algorithm. Through actual driving, this study’s researchers obtained images that correspond to general road conditions, harsh environments, distinct-shape vehicles, vehicles loading cargo, vehicles without visible patterns, and vehicles in tunnels, which are discussed in this paper primarily in accordance with the accompanying weather conditions (e.g., rainy, sunny, or cloudy). Common passenger vehicles, vans, trucks, and buses in the main lanes and side lanes of the acquired videos were used as positive samples. The road signs and windows that often cause misrecognitions in experiments were constructed as negative samples. In addition, the AdaBoost classifier can be composed with 15 layers, since the misrecognized results of the classifier can be reused as learning data for the training algorithm.

### Detection system

**Fig 2:**
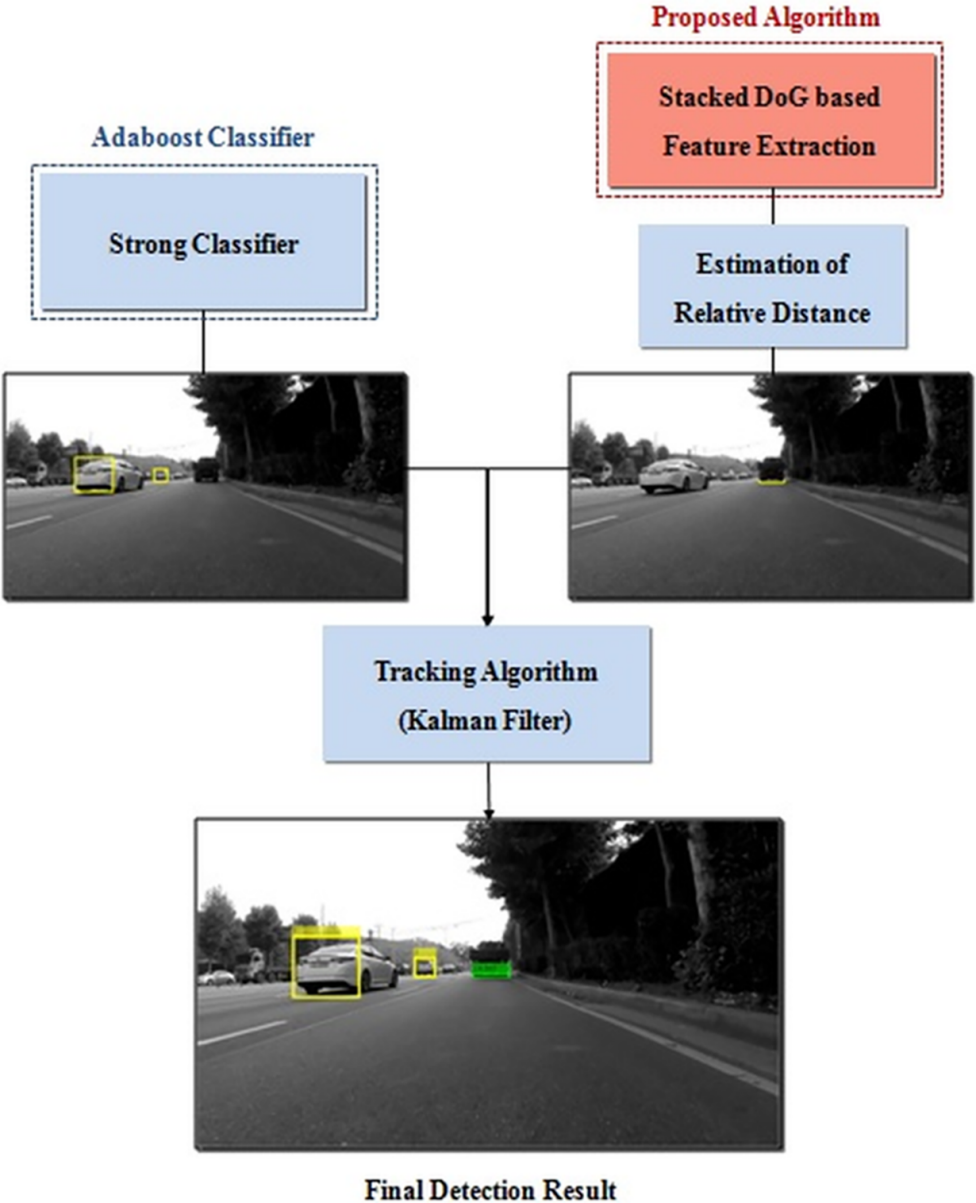
Structure of the detection system.

As shown in Fig 2, the new vehicle detection system is composed of an AdaBoost classifier, a distance estimation block, a tracking system, and the proposed stacked DoG-based feature extraction algorithm. The relative distances between the ego vehicle and its preceding vehicle are estimated from the ratio of the detected vehicle’s width in the frame to the actual width of the vehicle and focal length of the camera sensor [27–28]. The AdaBoost strong classifier is composed of various weak classifiers, and the weight of each weak classifier is adjusted to iteratively decrease classification errors [24]. This AdaBoost classifier detects target objects that exist in all traffic lanes that have vehicular appearance characteristics according to the training data. Even if the targets in the ego line lack the features of the training data, they can be recognized through the proposed feature extraction algorithm. The proposed algorithm specializes in detecting vehicles in the ego-line; however, the performance of proposed algorithm can be deteriorated when vehicles exist in other lanes; the Adaboost classifier is used to compensate for the situation. The detected results from the classifier and feature extraction algorithm are combined and entered into the tracking algorithm. If AdaBoost returns no recognition results within an ego line region in the images, the proposed algorithm is executed. Thus, a target that has could not be recognized by the AdaBoost method can be correctly detected by the proposed vehicle detection framework.

The locations of the detected vehicles are measurements of the Kalman filter in the tracking algorithm [5–7]. The Kalman filter estimates the location of detected vehicles in consecutive video frames, even under measurement noise [29–34].

## Stacked DOG-based feature extraction algorithm

**Fig 3:**
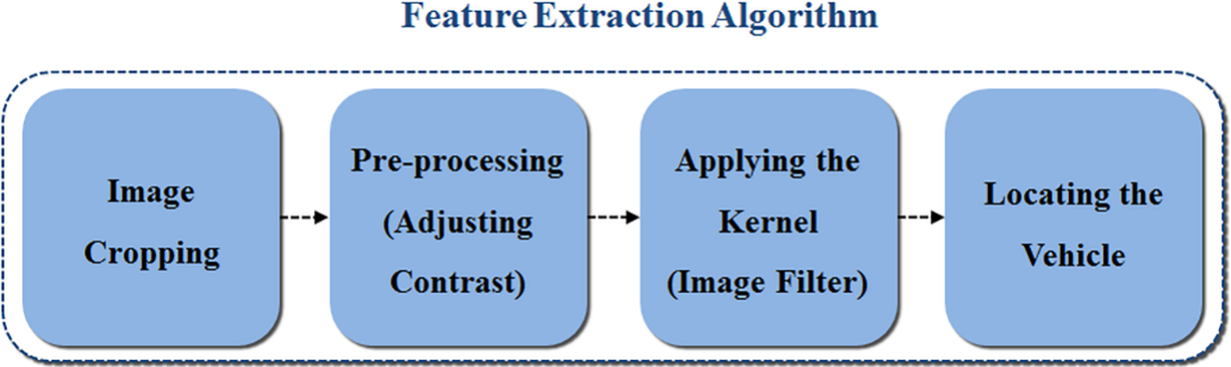
Structure of the feature extraction algorithm.

The proposed stacked DoG-based feature extraction algorithm extracts the shadow and rear wheels beneath a preceding vehicle in frames to recognize the exact location of the vehicle. The proposed algorithm is for vehicles that exist in the ego line, and vehicles ahead can be recognized without depending on the appearance of other vehicles. The proposed algorithm can be described as acting like active sensors similar to the radar in vehicle safety systems. The whole structure of the feature extraction algorithm is shown in Fig 3. The steps of the algorithm are image cropping, pre-processing adjusting contrast values, applying the kernel, and locating the vehicle. The detailed processes of each step are given in Sections 3.1–3.4.

### Image cropping

**Fig 4:**
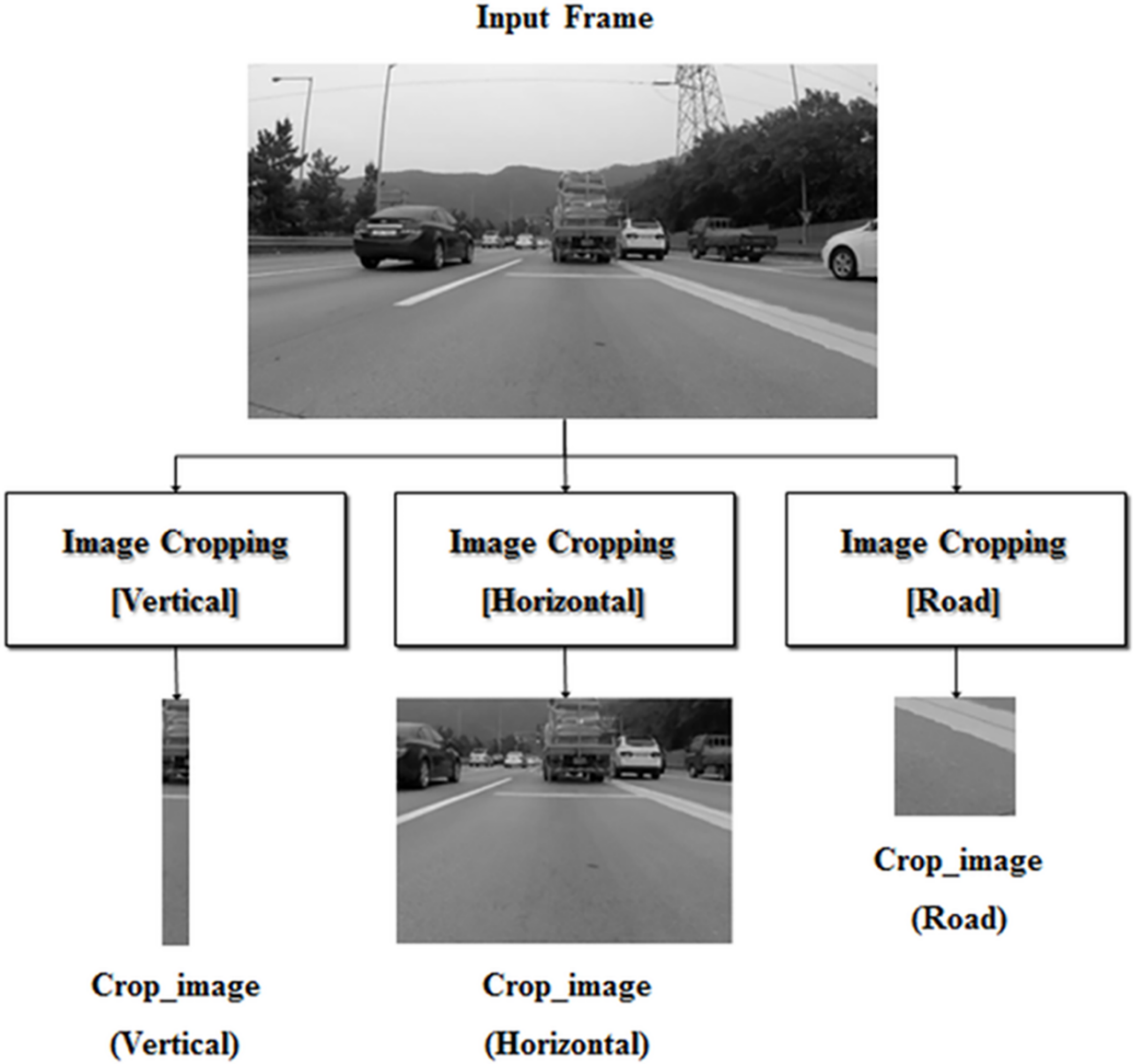
Image cropping.

In this step, the frame is cropped to locate the vertical and horizontal locations of a preceding vehicle. The proposed algorithm has to detect any vehicle in the ego-line; as such, image cropping is necessary to avoid misrecognizing vehicles in other lanes. As shown in Fig 4, the whole frame can be cropped into vertical, horizontal, and road image sections. The vertical and horizontal locations of vehicles can be determined using the vertical and horizontal images, respectively. The road image can also be extracted from video frames and used to recognize the illumination intensity in the road. The location and size of the cropped images are determined in proportion to the size of the frame.

### Pre-processing (adjusting the contrast value)

This step accentuates the regions of common features so that they can be recognized in frames, while also de-emphasizing other regions in the image. The shadow and rear wheels beneath a preceding vehicle are utilized as common characteristics in the algorithm; therefore, other regions of the image are considered unnecessary in the detection process. In a worst-case scenario, these can lead to the misrecognition of a preceding vehicle. In order to avoid this, feature locations and de-emphasized unnecessary regions by adjusting the image’s contrast value. The different intensity values between the characteristic region and the road region are obvious, so common feature areas have larger contrast values than other regions.

**Fig 5:**
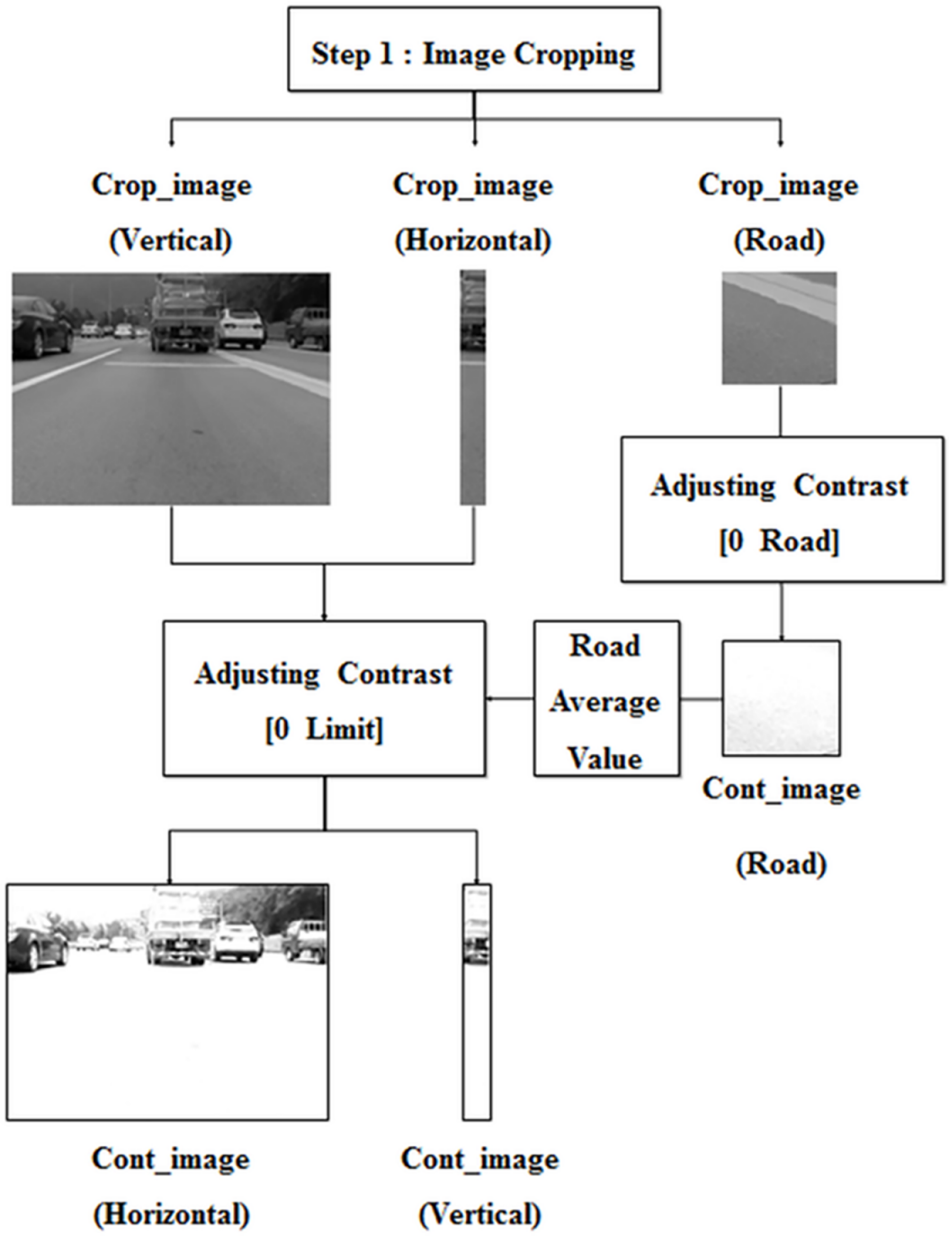
Pre-processing (adjusting the contrast value).

Thus, as shown in Fig 5, the region of features can be accentuated by applying certain contrast values to the image. This contrast value (Limit) is determined in proportion to the average pixel intensity in the road image from the first step; higher average pixel values require brighter road illumination and higher contrast values. The road image is pre-processed (Cont_image) to calculate the average value and avoid the effects of shadows and road signs; thus, this step eliminates horizontal and vertical images with adjusted contrast values.

### Applying the stacked DoG kernel

In this step, the proposed stacked DoG kernel is applied to the horizontal and vertical images from the second step. This edge detection method has been utilized in many research studies to determine the horizontal and vertical lines of preceding vehicles [15,17,21]. When using this method, all edge components—including regions unrelated to a vehicle—are clearly displayed in the image, which can lead to misrecognition in the vehicle detection algorithm.

**Fig 6:**
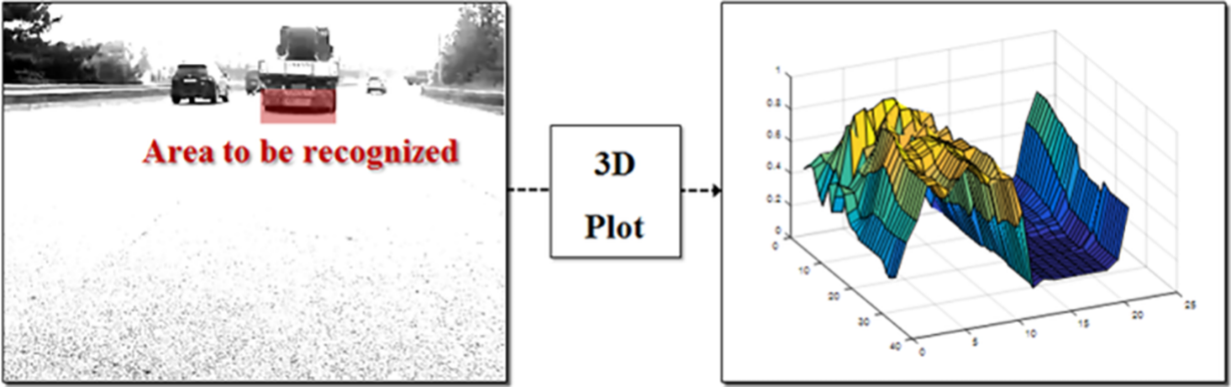
Result of a 3D plot in the region to be recognized.

However, when using the kernel and applying this to frames via an image convolution method, only certain regions that have a similar pattern to the used kernel have strong filtering responses in the image. Thus, specific kernels are composed that have a similar pattern to areas to be recognized. The region to be recognized is the location of the shadow and rear wheels beneath preceding vehicles. Thus, as shown in Fig 6, we plot this area in the image to determine the proper kernel to use.

**Fig 7:**
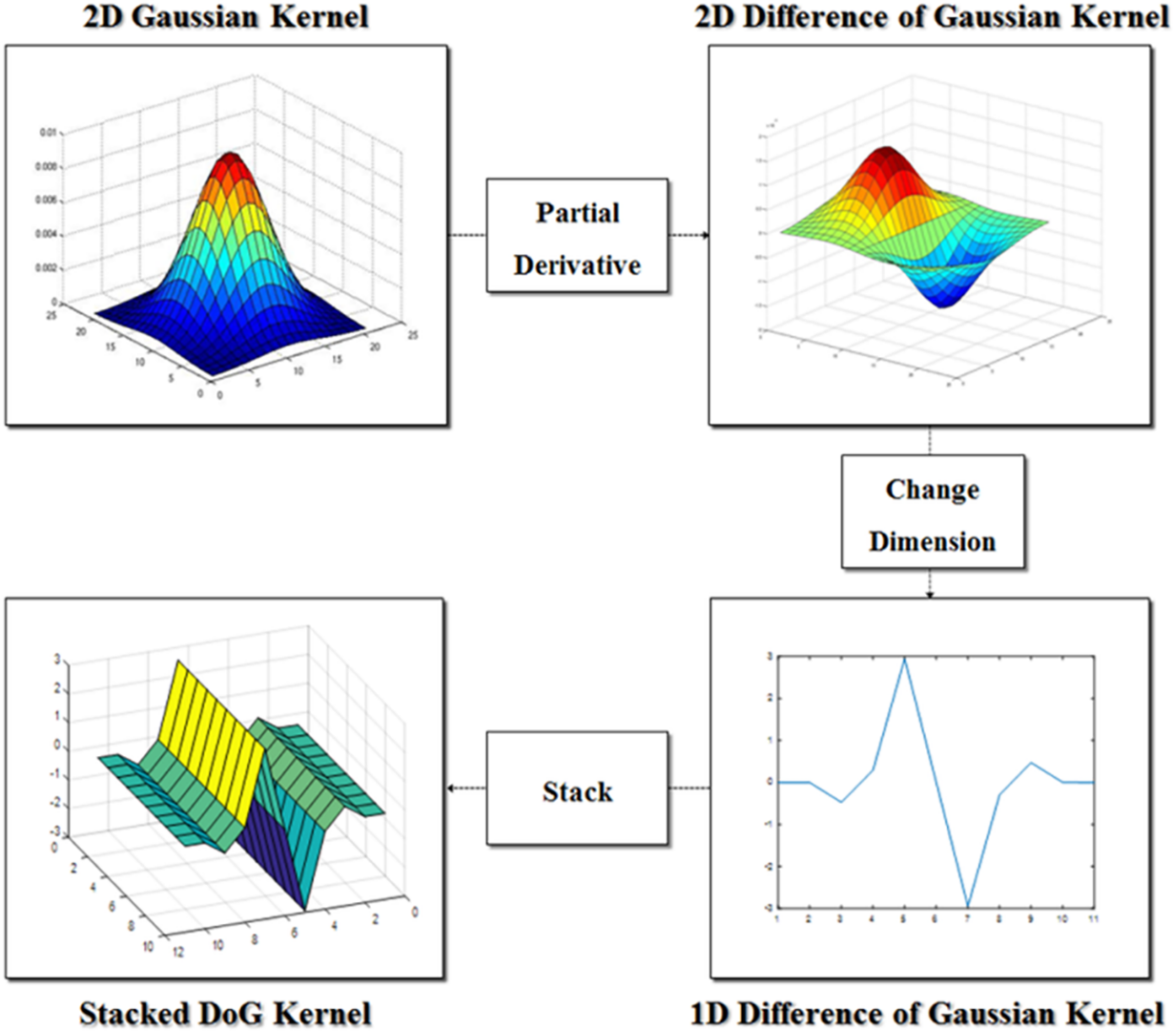
Process of composing a stacked DoG kernel.

A Gaussian filter is utilized to smooth and suppress noise in the image; this is usually added to the pre-processing step when detecting the edge components in the frame. The shape of the two-dimensional Gaussian and Difference of Gaussian (DoG) filter is shown in Fig 7. The section of the Difference of Gaussian kernel has a similar shape to the pattern underneath the vehicle in the frame. However, instead of applying one- and two-dimensional DoG kernels, a new kernel composed by stacking the one-dimensional Difference of Gaussian kernels in the y-direction is applied to eliminate false recognition from increasing the difference in the filtering response value of one- and two-dimensional DoG. As a result, this kernel is called a stacked DoG.

**Fig 8:**
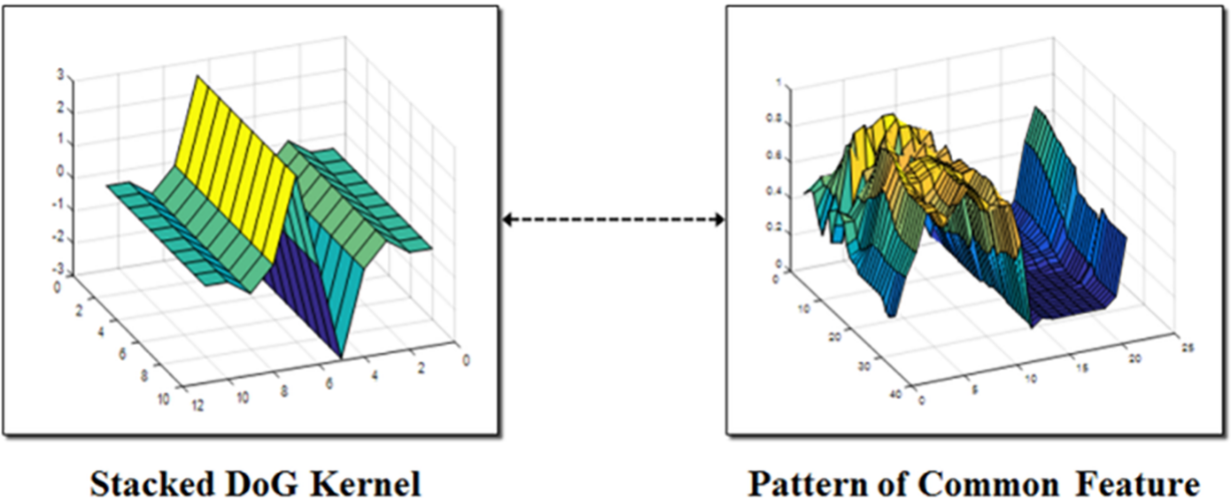
Comparison of the stacked DoG and patterns of the feature.

As shown in Fig 8, the stacked DoG kernel and the pattern in the region of common features have similar shapes. This kernel is applied to the frame using an image convolution method, and the used stacked DoG kernel is scaled proportionally to the length of the vertical location of the preceding vehicle, since the vehicle width is almost proportional to the vertical position of the vehicles in the frames. Then, the determined kernel window is applied to the upper left and slides to the lower right of the image for every pixel.

**Fig 9:**
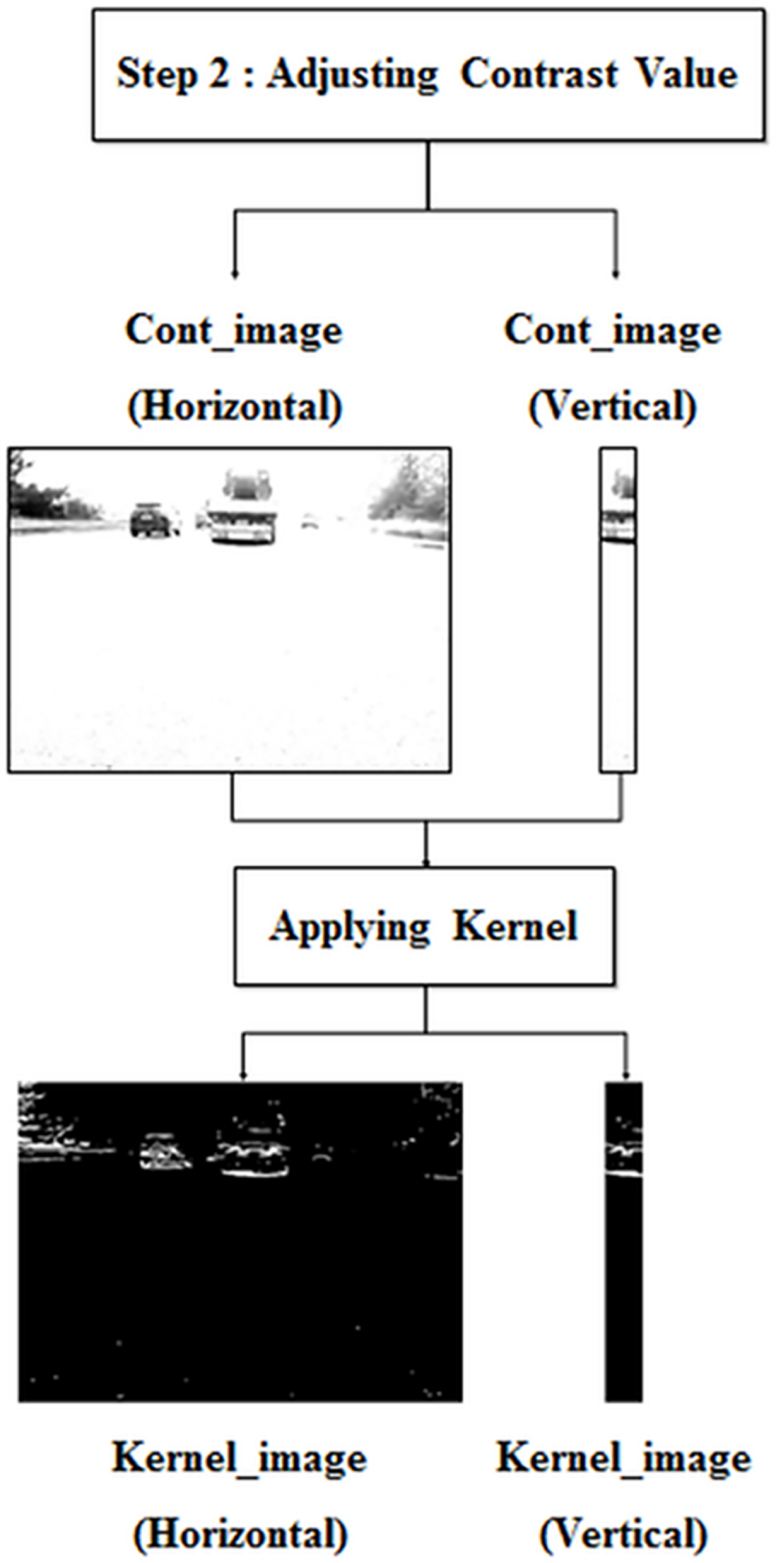
Applying the kernel.

As mentioned above, the values of the filtering response in a specific area have similar values to the kernel window and are larger than in other areas. Thus, the filtering response in the area of common features is stronger than in the other areas, as shown in Fig 9. The exact vertical and horizontal location of a preceding vehicle can be detected in the next step based on this filtering response.

### Finding the location of a vehicle

In this step, the exact location of features is recognized through the filtering response in the third step. First, the sum of the filtering responses is calculated for each row in a vertical image. Then, the vertical location of the vehicle is recognized by finding the index, which has a filtering response that exceeds a threshold for the first time. This threshold is obtained by finding the average of all peak values rather than using the largest peak value because this value is sometimes in the filtering response in the bumper or windshield of a vehicle that has a strong horizontal characteristic. Thus, if the filtering response of a certain index is larger than the threshold, this index is applied to the vertical location of the vehicle (index_ver). The right and left indices of the vehicle can be recognized by creating a search region based on the determined vertical location of the vehicle. In this region, the leftmost (index_left) and rightmost (index_right) indices that have a specific filtering responses are respectively applied to the left and right positions of the vehicle.

## Experiments

**Fig 10:**
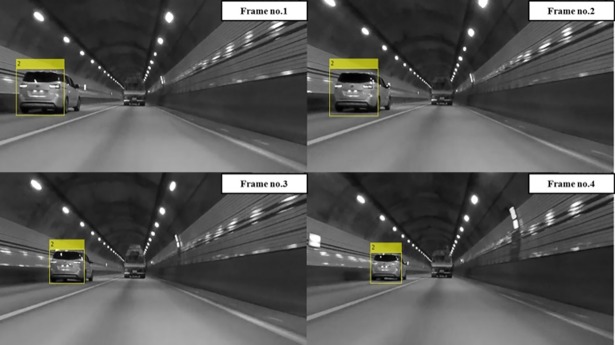
Experimental results using the original AdaBoost classifier.

**Fig 11:**
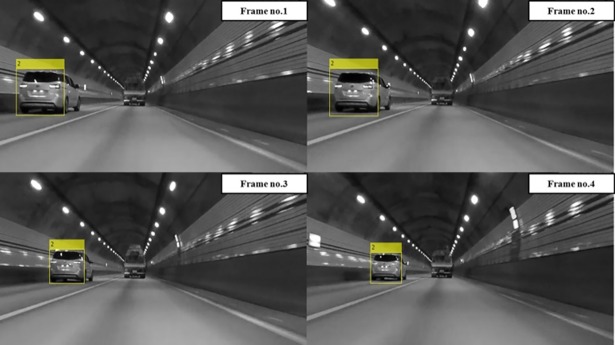
Experimental results using the detection and tracking part of FCAAS.

Experiments were conducted by installing an optical sensor at the front of the vehicle. The optical sensor used was a GoPro Hero4 Session, 720 p, 30 fps, with a medium field of view (FOV) that provided 1280x720 frames. The obtained frames were processed in Matlab® on an Intel® Core™ i5 3.20 GHz processor with 8.00 GB RAM. The average processing time for the proposed detection system was approximately 0.15 seconds per frame compared to a processing time of about 0.1 seconds when using the original AdaBoost classifier. In addition, the vehicle detection and tracking processing time of the forward collision avoidance assist system (FCAAS) algorithm with the AdaBoost + particle filter method [35], was approximately 0.2 seconds. From these results, it was recognizable that there was little difference in the amount of computation in the proposed detection system. In Figs 10 and 11, the results of the AdaBoost-based system classifier are displayed in the yellow box, and the results of the proposed stacked DoG-based feature extraction algorithm are displayed in the colored box underneath the vehicles. The color of the box varies according to the relative distance so that the distance between the ego vehicle and the preceding one can be easily recognized. Precision is calculated as follows:

$$precision=\frac{Number\;of\;true\;positives}{Number\;of\;positives}$$ (1)

The AdaBoost-based algorithm recognizes targets based on the appearance of preceding vehicles. Thus, there are several harsh environments in which vehicles cannot be detected using the original AdaBoost classifier and the detection and tracking parts of the FCAAS [35]. These harsh environments are largely divided into two cases. First, vehicles that have an unusual shape or are transporting a large cargo cannot be detected. Second, harsh environments may make the patterns or shapes of preceding vehicles invisible in video frames. The second case typically occurs when preceding vehicles are too far from the optical sensor to recognize the pattern, or when the pattern of the preceding vehicle cannot be recognized because of its dark color or the light conditions of the road.

**Fig 12:**
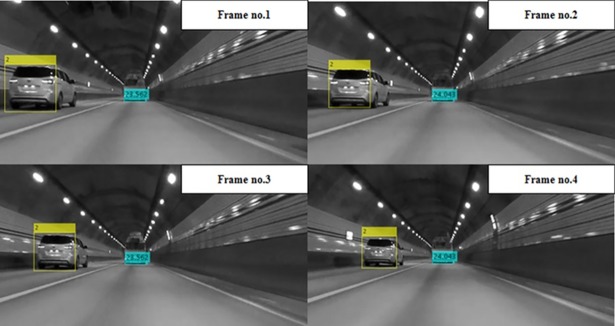
Experimental results using the proposed detection system.

In general cases, the AdaBoost-based algorithm can recognize preceding vehicles well, but as shown in Figs 10 and 11, preceding vehicles in harsh environments cannot be recognized using those algorithms. However, the preceding vehicle can be correctly recognized and tracked in the video frames as shown in Fig 12 when using the proposed vehicle detection system.

Precision sharply decreases in harsh environments when using the original AdaBoost classifier and the AdaBoost+particle filter method of FCAAS [35] as shown in Table 1. The average precisions of the original AdaBoost classifier and the AdaBoost + particle filter method are 59.2% and 48.8%, respectively. Meanwhile, the proposed detection system is capable of attaining the highest average precision of 93.7%, even in harsh environments. Precision is generally low in challenging light conditions because the AdaBoost classifier in the vehicle detection system is affected by the road’s light conditions. The biggest factor in reducing the proposed detection system’s recognition rate is that the AdaBoost classifier sometimes does not recognize vehicles in the side lane because the perspective on vehicles in the side lane differs from that of preceding vehicles. The precision of the proposed algorithm is confirmed as being improved compared to the other two algorithms, as shown in Table 1.

**Table 1:** Comparison of precision under some harsh environments.

	Cases	Proposed	FCAAS	AdaBoost
Appearance	Distinct Shape Vehicles	97.5%	68.3%	59.9%
	Vehicle Loading Large Cargos	95.8%	65.1%	53.4%
Light	Vehicles with No Visible Pattern	93.9%	38.5%	25.5%
	Vehicles in Tunnel	87.4%	64.8%	56.3%
Average		93.7%	59.2%	48.8%

**Fig 13:**
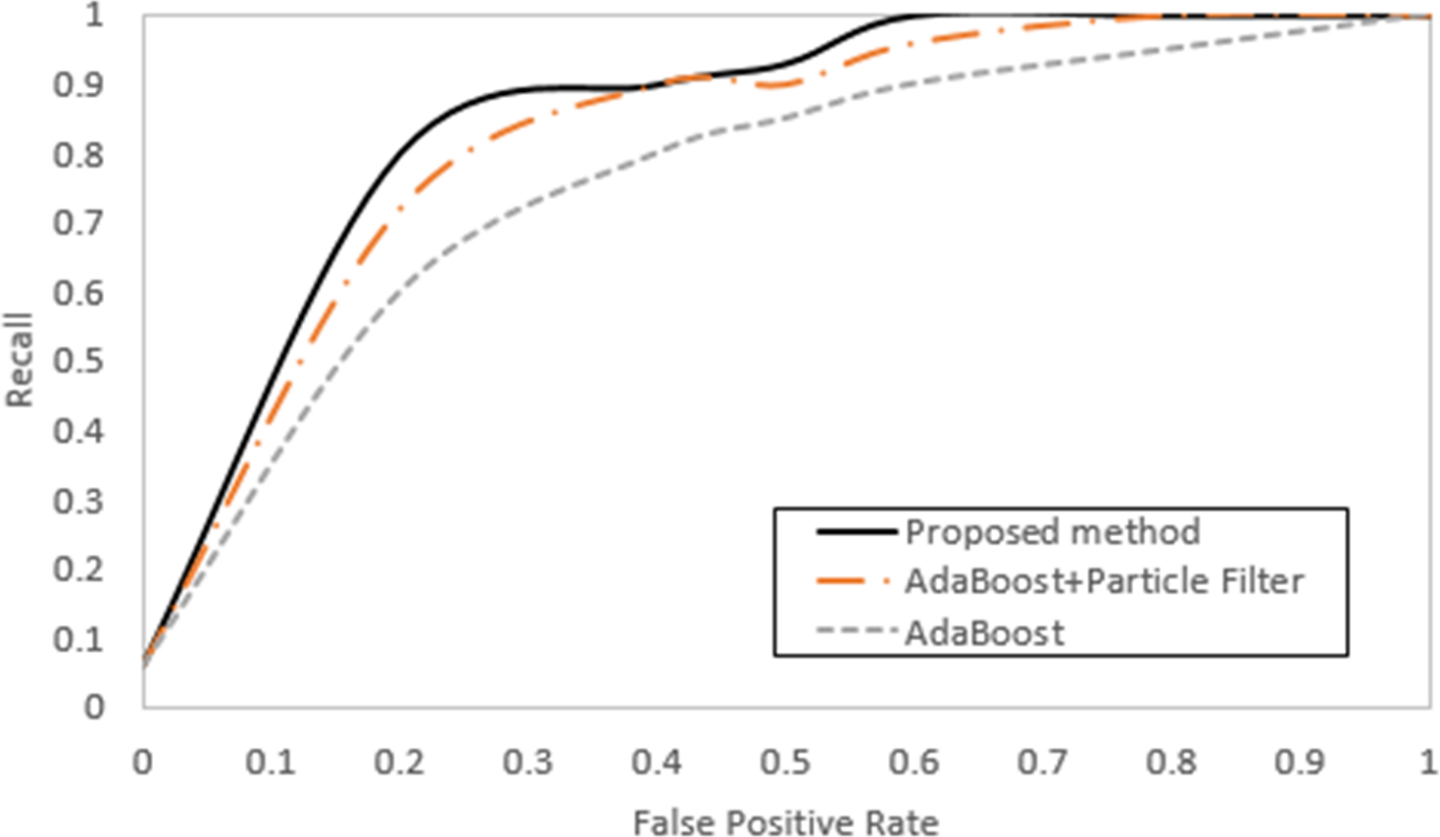
ROC’s of the three detection methods.

Fig 13 shows the receiver operating characteristic curves (ROCs) of the proposed method, the FCAAS algorithm, and the original AdaBoost method. The Y-axis represents the average number of true positives (recall) detected in one image and the X-axis represents the number of false positives per frame. In the results of the ROCs, the proposed method has the highest recall at the same false positive rate (FPR), meaning that the number of true positives is larger than for the other two algorithms. This performance difference is most noticeable in harsh conditions.

### Case 1: Distinct shaped vehicles

**Fig 14:**
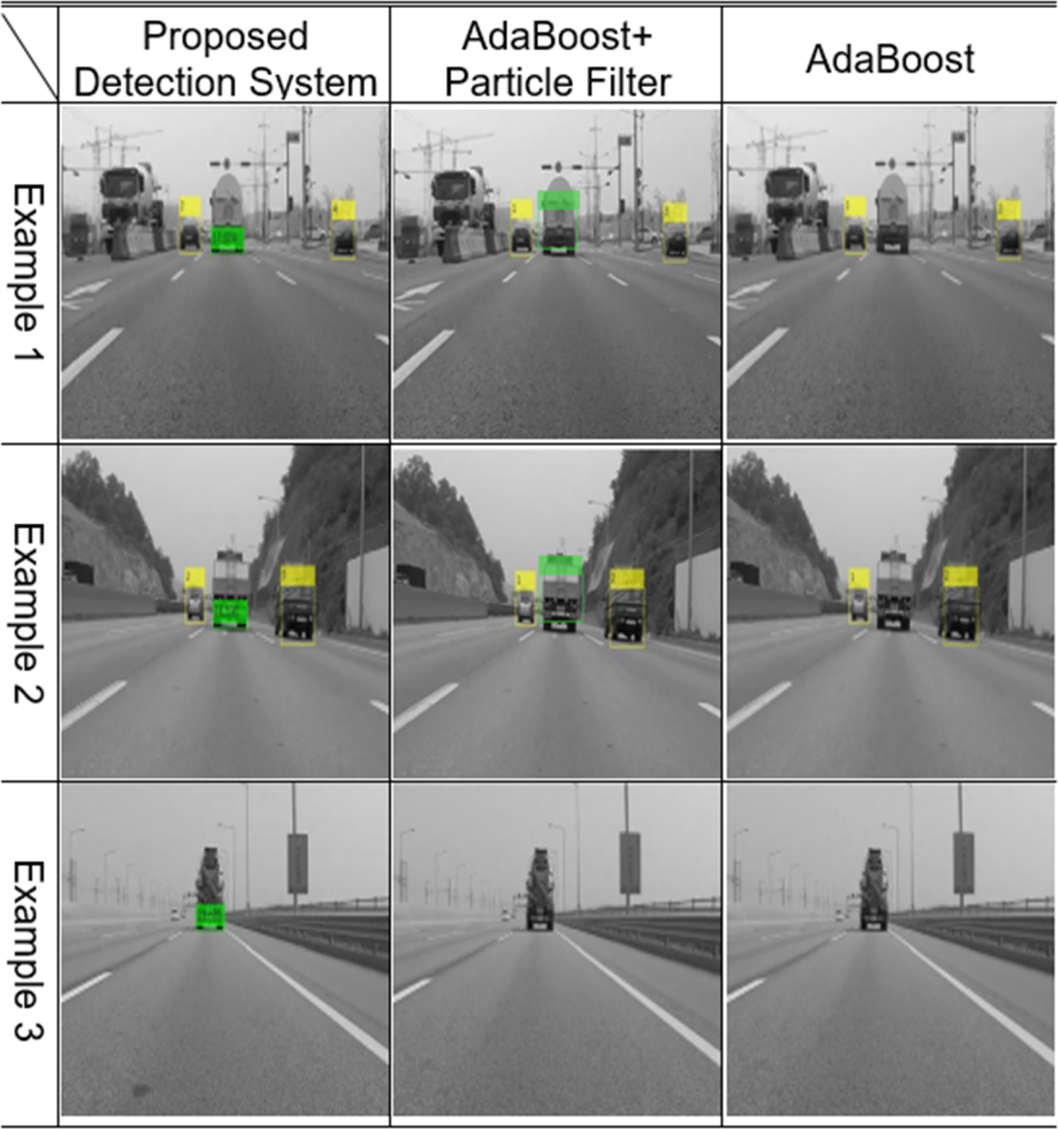
Experimental results: Distinct-shaped vehicles.

As shown in Fig 14, if the shapes of preceding vehicles differ completely from the general shapes of vehicles in the training data, the detection and tracking aspect of FCAAS [35] cannot recognize them. Supplementing the training data for such vehicles into the learning algorithm solves this problem. However, detection failures will occur continuously each time a new differently shaped vehicle appears. The proposed algorithm recognizes the common characteristics of vehicles, so any vehicles that exist in the ego line that have this feature can be correctly recognized as follows.

### Case 2: Distinct-shaped vehicles

**Fig 15:**
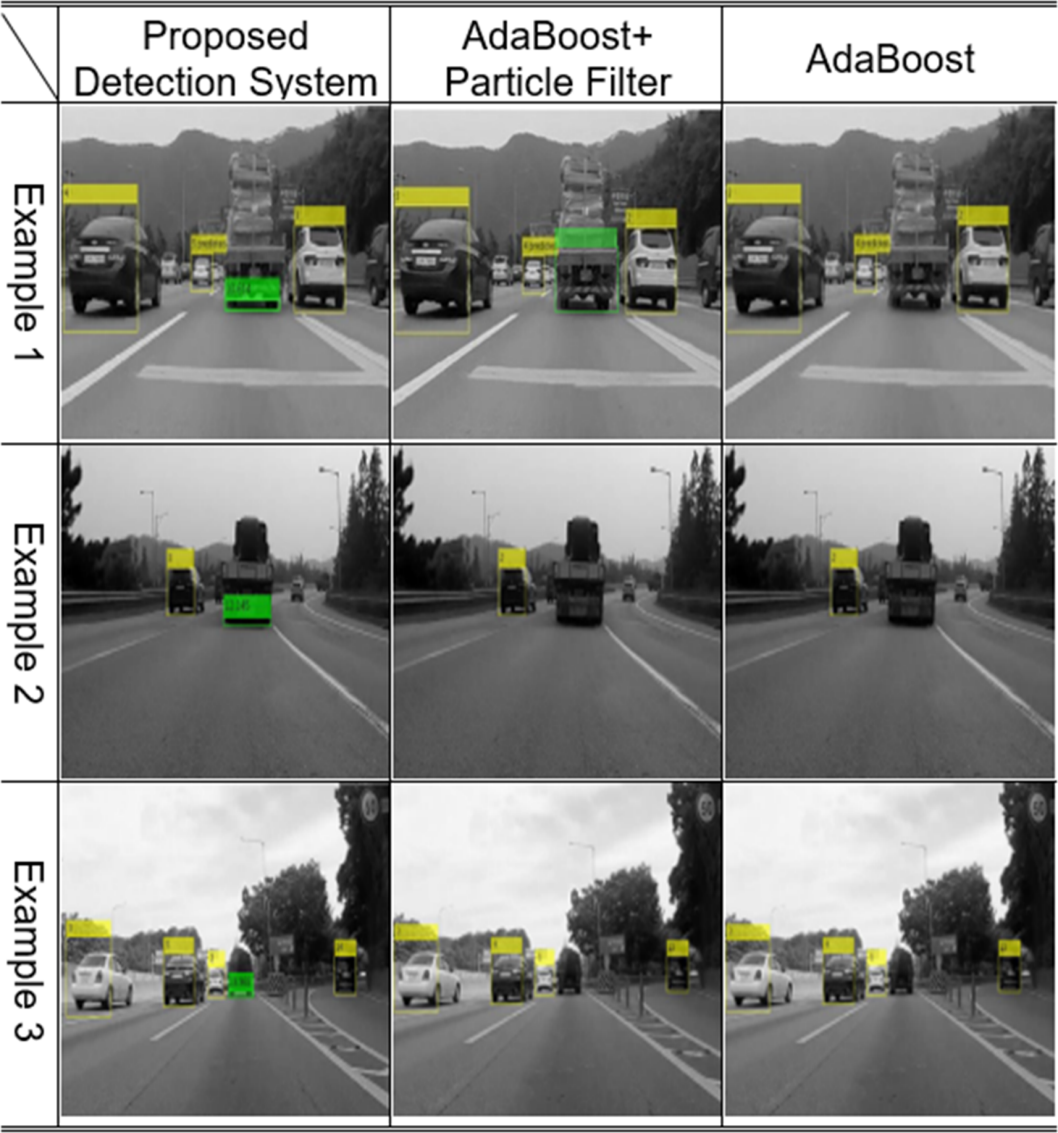
Experimental results: Vehicles loading large cargos.

If a preceding vehicle has a large cargo load, its shape and pattern will completely change, and detection failures can occur in such cases. However, no matter how much luggage is loaded into a vehicle, the pattern of the shadow and rear wheels underneath the vehicle does not change. Thus, such vehicles can be precisely recognized using the proposed detection system as shown in Fig 15.

### Case 3: Vehicles without visible patterns

**Fig 16:**
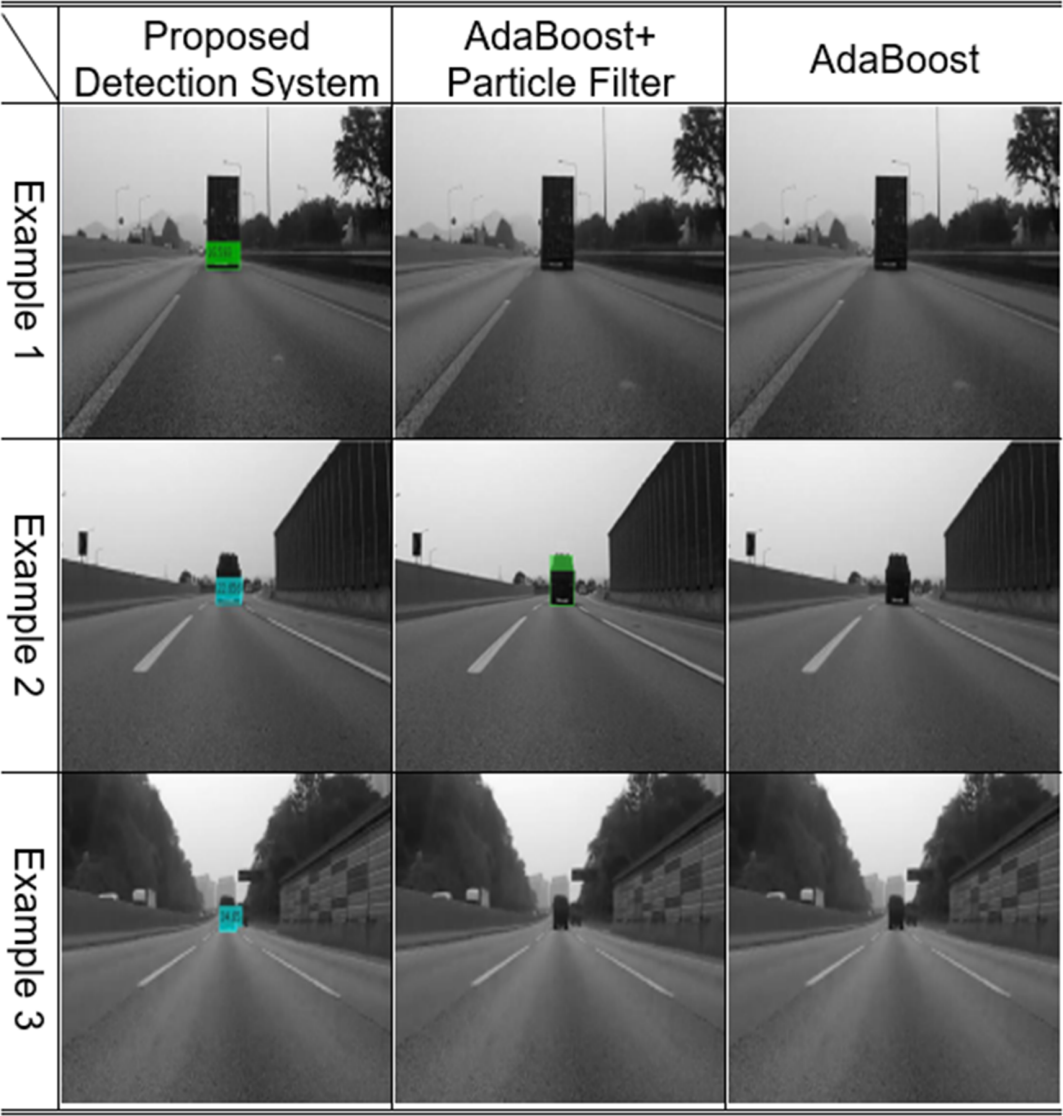
Experimental results: Vehicles without visible patterns.

If the inner patterns of vehicles cannot be seen because of the light conditions, such vehicles will never be recognized by AdaBoost because this method depends on the appearance and pattern of the target. Situations in which the pattern becomes invisible are as follows: First, if the pattern of the preceding vehicles cannot be seen due to backlights in traffic. Second, the pattern may not be visible for dark-colored vehicles. The final situation is when the vehicles in front are far from the vision sensor. These far-off preceding vehicles cannot be detected by the AdaBoost-based vision systems. If vehicles are not recognized in the frame, their relative distance cannot be estimated, necessitating the use of an active sensor such as a radar in detection systems. However, since even distant vehicles have common features, they can be recognized using the proposed algorithm. Vehicles without visible patterns due to various factors can be detected with the proposed stacked DoG-based feature extraction algorithm, as shown in Fig 16. Therefore, the limitations of vision sensors can be complemented through this approach.

### Case 4: Vehicles in a tunnel

**Fig 17:**
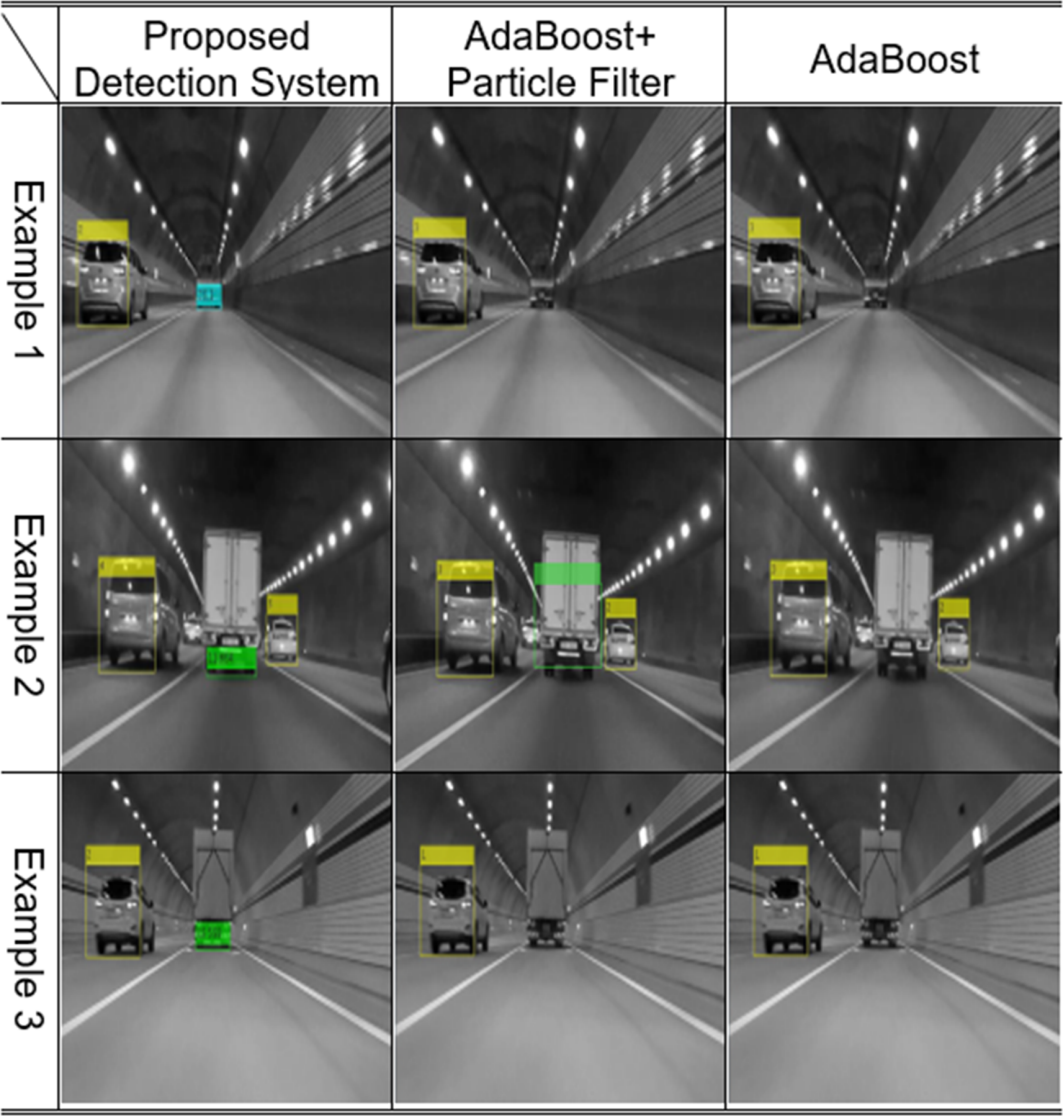
Experimental results: Vehicles in a tunnel.

Failures to detect preceding vehicles often occur in tunnels, since the light conditions change the patterns of preceding vehicles. Distinct-shaped vehicles in a tunnel are often not recognized by the AdaBoost-based algorithm. However, even in these varied light conditions, the common characteristics of the shadow underneath and the rear wheels of vehicles remains unchanged, enabling the detection of any vehicles in the ego-line by the proposed algorithm, as shown in Fig 17. Vehicle detection technologies are based on vision sensors for preceding vehicles, thus the increased recognition rate for vehicles in such situations is meaningful.

As shown in these figures, vehicles in harsh environments cannot be recognized using AdaBoost-based methods. The precision of algorithms based on AdaBoost rapidly decreases when the shape or pattern of a vehicle is outside the boundaries of the training data for various types of vehicle and road conditions. These phenomena are fatal in the implementation of vehicle detection systems in the field, because they lead to decreased reliability in FCW, AEB, and smart cruise control technologies; ultimately, malfunctions in such systems can threaten human safety. However, the proposed detection system maintains high precision in these harsh environments, ensuring the system’s reliability. In addition, far-off preceding vehicles are not detected by AdaBoost vision systems since their shapes and patterns cannot be seen in the frame. If vehicles are not recognized in the frame, their relative distance cannot be estimated. However, it is possible to precisely recognize the location of far-off preceding vehicles using the proposed detection system with only the optical sensor. The limitation of the recognizability distance may be increased by altering the size and shape of the image frame. In addition, the contours of a preceding vehicle can be precisely detected by the proposed detection system even if a vehicle is moving quickly on the road, which is important because the measured distance of a preceding vehicle can only be accurate if the contours of the vehicle are accurately recognized.

Thus, any vehicles in the ego lanes can be recognized under various road conditions using the proposed vehicle detection system, and estimations of the relative distance from a preceding vehicle can be more accurately conducted by recognizing the exact contours of preceding vehicles. In the future, the reliability of the detection system can be enhanced by improving the vision sensors.

## Conclusions

In this paper, a novel vehicle detection system was presented that increases the reliability of vehicle safety technology based on vision sensors. The system was combined with the AdaBoost classifier and the novel stacked DoG-based feature extraction algorithm. The proposed algorithm could detect preceding vehicles that exist in the ego line, not by their appearance or pattern, but rather by common features that all vehicles share. This enabled the detection of the location of preceding vehicles that AdaBoost-based methods could not find, lending credence to the theory that the proposed algorithm could overcome the existing problems of vehicle detection systems. In addition, the contours of a preceding vehicle far from the ego vehicle could be accurately detected using the proposed algorithm. This may lead to the more accurate recognition of vehicles’ relative distance in detection systems based on optical sensors. Thus, the proposed vehicle detection system is expected to be widely used in automotive safety systems to detect preceding vehicles and estimate relative distances. Although tracking algorithms can be considered in various ways, the filter-based tracking algorithm can be narrowed into two types: infinite impulse response filter- and finite impulse response filter-based tracking. Applying these filtering algorithms could improve the proposed algorithm and is assumed to be a future research direction.
